# Antenatal predictors of stem cell content for successful umbilical cord blood donation

**DOI:** 10.1007/s00404-021-05970-7

**Published:** 2021-02-15

**Authors:** Anna Funk, Johanna Buechel, Evelyn Annegret Huhn, Doris Mueller, Cristina Granado, Dimitrios Tsakiris, Andreas Schoetzau, Jakob Passweg, Irene Hoesli, Gwendolin Manegold-Brauer

**Affiliations:** 1 Department of Obstetrics and Antenatal Care, University Hospital Basel, Basel, Switzerland; 2 Department of Obstetrics and Gynecology, Ludwig-Maximilians-University, Maistrasse 11, 80337 Munich, Germany; 3 Department of Hematology, University Hospital Basel, Basel, Switzerland; 4 Department of Biomedicine, University Hospital Basel, Basel, Switzerland

**Keywords:** Public cord blood banking, Total nucleated cell count, Hematopoietic stem cell transplantation, Prospective single center study, Multivariate analysis

## Abstract

**Purpose:**

The most important HLA-independent factor for the selection of cord blood units (CBU) for hematopoietic stem cell transplantation is the total nucleated cell (TNC) count over 150 × 10^7^ as a surrogate marker for stem cell content. The purpose of this prospective study was to define prenatal clinical predictors for TNC count that would help to identify successful CBU donors before the onset of active labor.

**Methods:**

This was a prospective analysis of 594 CBUs, collected from all eligible term singleton pregnancies at Basel University Hospital between 4/2015 and 9/2016 analyzing several maternal and fetal factors. The impact of these factors on TNC count (< 150 × 10^7^ cells vs. ≥ 150 × 10^7^ cells) of the CBUs was modeled in a multivariate analysis.

**Results:**

A total of 114 (19.2%) CBUs had a TNC count of ≥ 150 × 10^7^. In a ROC analysis there was no significant difference between the AUC of all prenatal factors (AUC 0.62) and estimated fetal birth weight by ultrasound alone (AUC 0.62). For women planning a trial of labor a recruitment cut-off at an estimated birth weight of 3300 g would allow 72.6% of all donors with sufficient TNC count to be recruited and 22.8% of all collected CBUs would have a sufficient TNC count for banking. For women planning for elective CS a cut-off of 3400 g would allow 71.4% of all donors with sufficient TNC count to be recruited and 22.7% of all collected CBUs would have sufficient TNC count for banking.

**Conclusion:**

The estimated fetal birth weight within 2 weeks of delivery by ultrasound as single parameter can be considered at the time of recruitment to estimate the chances of a successful CBU donation.

## Introduction

Once considered a waste product discarded with the placenta, umbilical cord blood is now commonly used as a source of potentially life-saving hematopoietic stem cells used in the treatment of malignant and nonmalignant hematologic and immunologic diseases in the absence of a matched sibling or unrelated donor [1]. On the basis of average family size, less than 30% of patients will have a matched sibling donor. Following the first successful umbilical cord blood transplantation in 1988, unrelated public cord blood banks were established. An estimated 700,000 CBUs have been donated and stored to date and over 40,000 CBU transplants have been performed worldwide [2]. Over 25,000 patients have been cured with this approach [3, 4].

Compared to hematopoietic stem cells from bone marrow, CBU has several advantages: rapid accessibility due to the possibility of cryopreserving fully tested and human leucocyte antigen (HLA)-typed units, lowered risk of and less severe acute and chronic graft-versus-host disease, reduced likelihood of transmitting infections, increased ethnic representation and risk- and pain-free collection [5]. The most important disadvantage of umbilical cord blood is the limited number of stem cells in the CBU that typically are around ten times lower than in bone marrow. So only units with a sufficient count will be used for hematopoietic stem cell transplantation [6].

The most important HLA-independent factor for the selection of a CBU for hematopoietic stem cell transplantation is the total nucleated cell count (TNC) as a surrogate marker for stem cell content [6]. At present, about one in five donors can provide a CBU with a sufficient TNC count (≥ 150 × 10^7^). The efforts to obtain consent of all eligible donors are high and optimization of the selection is needed. Several parameters of the mother, the neonate, the course and mode of delivery have a direct impact on TNC count in the CBU [7, 8]. However, intrapartum factors cannot be used for donor selection since recruitment needs to take place before the onset of active labor.

Newborn weight seems an important factor consistent in previous studies [7, 9–12]. However, sonographic fetal weight estimation before delivery has a standard error of around 7% and to date there have been no studies regarding the value of estimated fetal weight instead of newborn weight. The purpose of this study was to perform a prospective single center study with the aim of developing a simple and effective selection algorithm for recruitment of CBU donors for public banking that would help to identify successful CBU donation with a high TNC count before the onset of labor.

## Materials and methods

### Study design

This was a prospective study of 594 CBUs, collected between April 2015 and September 2016 at the delivery unit, University Hospital Basel, Switzerland. All women eligible for CBU donation were asked to participate in the study. Inclusion criteria were mothers with singleton term pregnancies (> 37 + 0 gestational weeks) without exclusion criteria based on medical history and donor questionnaire.

The following factors were considered: maternal factors were age, gravidity, parity, weight at the beginning of pregnancy, height and planned mode of delivery. Neonatal factors were sex, estimated fetal weight by ultrasound according to Hadlock formula [13] (abdominal circumference; biparietal-diameter; femur length) within 2 weeks of delivery and sonographic diameter of the umbilical cord vein (mm).

Every woman entering the delivery unit that did not have exclusion criteria from their medical history was considered as a potential donor to the public CBU bank. After consenting and answering a donor selection questionnaire based on international criteria for CBU banking, the CBUs were collected according to the guidelines edited by the Foundation for the Accreditation of Hematopoietic Cellular Therapy (FACT) with standard operating procedures. After clamping of the umbilical cord between 30–60 s after delivery, collection was conducted under sterile conditions into a sterile collection bag (MacoPharma, Switzerland; Fenwal Europe, Belgium). Within 6 h the collection bag was sent to the stem cell laboratory located on hospital grounds for further processing.

An autoanalyzer (ADVIA 2120, Bayer, Basel, Switzerland) was used to count the TNCs upon arrival of the CBU at the stem cell laboratory. If the TNC count was ≥ 150 × 10^7^ the CBU was kept for further processing, done within 36 h after collection, storage and registration in the Swiss public registry. To reduce volume an automated method was performed (Sepax, Biosafe SA, Switzerland). Cells were supplemented with 7.5% dimethyl sulfoxide as cryoprotectant and deep frozen by controlled-rate freezing. The samples were then cryopreserved in liquid nitrogen for storage at − 196 °C. In cases of a TNC count below 150 × 10^7^ the CBU was discarded after analysis of TNC according to standard protocols.

### Statistical analysis

All factors known prior to delivery were analyzed in regard to prediction of TNC count. Maternal factors were age, gravidity, parity, height, weight at the beginning of pregnancy and at delivery as well as the planned mode of delivery. Neonatal factors included sex, estimated fetal weight by ultrasound according to Hadlock formula and sonographic diameter of the umbilical cord vein (mm).

The study population was divided into two subgroups: TNC count of less than 150 × 10^7^ cells versus TNC count of at least 150 × 10^7^ cells. Since the mode of delivery is also known to have an influence on the content of CBU, analysis was done by planned mode of delivery: we analyzed women who planned to have an elective cesarean section (CS) separately from women who planned a trial of labor. For women with planned CS the mode of delivery was integrated as a known factor prior to delivery.

Descriptive statistics are presented as counts and frequencies for categorical data and median and min, max for ordinal or metric data. *p*-values correspond to Mann–Whitney *U* test (for medians) and in case of categorical variables Chi-squared or exact Fisher test when the expected frequencies were less than five.

All factors known prior to delivery were analyzed in regard to prediction of TNC count. To predict a TNC count of ≥ 150 × 10^7^ a multivariable logistic regression was done. Subsequent ROC curves with corresponding AUC values were estimated. In order to prevent overfitting, estimation and comparisons of AUC`s were done using internal ten times tenfold cross validation. AUC is reported as the mean over all eight cross validations. Validation and imputation was done using the package "caret" within the software package R as described [14]. This is a suitable package to validate regression models. A *p* value < 0.05 was considered as significant. All evaluations were done using the statistical software R version 3.1.1. [15].

### Ethical approval

The study was approved by the Ethics Committee Northwest/Central Switzerland (EKNZ 2014–319), the responsible regional Ethics Committee based in Basel and part of Swissethics. All women gave written consent for CBU donation and for the study.

## Results

Between April 2015 and September 2016, a total of 594 CBUs were collected for this prospective study. The mean TNC count within the study group was 95.8 × 10^7^ cells. The mean weight of collection bags was 122 g. A total of 114 (19.2%) CBUs had a TNC count of ≥ 150 × 10^7^. The majority of women (*n* = 532) planned a trial of labor while 62 women planned to have an elective CS (10.4%) (Table 1). We analyzed the spontaneous labor group (*n* = 532) and the planned CS group (*n* = 62) separately.

**Table 1:** Baseline characteristics of recruited patients

	All	TNC < 150 × 10^7^	TNC ≥ 150 × 10^7^	*n*	*p* value
*n*	594	480	114		
TNC × 10^7^	95.8 [8.20; 373]	83.8 [8.20; 150]	182 [150; 373]	594	
Paritiy				594	0.065
0	336 (56.6%)	259 (54.0%)	77 (67.5%)		
1	192 (32.3%)	163 (34.0%)	29 (25.4%)		
2	51 (8.6%)	44 (9.2%)	7 (6.1%)		
3	15 (2.5%)	14 (2.9%)	1 (0.9%)		
Maternal weight (kg)	77.0 [47.0; 138]	76.0 [47.0; 138]	78.2 [55.0; 138]	565	0.101
Maternal height (cm)	167 [103; 186]	167 [103; 185]	168 [152; 186]	572	0.464
Estimated fetal weight (g)	3400 [2230; 4500]	3360 [2230; 4500]	3500 [2700; 4300]	546	< 0.001
Abdominal circumference (cm)	34.0 [27.0; 48.4]	33.9 [27.0; 39.5]	34.5 [30.1; 48.4]	490	< 0.001
Head circumference (cm)	33.3 [28.0; 38.0]	33.3 [28.0; 37.0]	33.1 [31.0; 38.0]	465	0.588
Femur lenght (cm)	7.30 [4.00; 8.40]	7.30 [4.00; 8.40]	7.32 [6.10; 8.30]	489	0.03
Diameter of umbilical vein (mm)	8.70 [5.00; 15.9]	8.70 [5.00; 15.9]	8.80 [6.20; 12.7]	316	0.387
Pregnancy week (days)	280 [255; 296]	280 [255; 296]	282 [265; 293]	594	0.007
Fetal sex				594	0.283
Male	316 (53.2%)	261 (54.4%)	55 (48.2%)		
Female	278 (46.8%)	219 (45.6%)	59 (51.8%)		
Planned mode of delivery				594	< 0.001
Trial of labor	532 (89.6%)	426 (80.1%)	106 (19.9%)		
Elective CS	62 (10.4%)	54 (11.2%)	8 (7.02%)		
Weight of collection bag (g)	122 [34.5; 234]	118 [34.5; 190]	153 [118; 234]	594	< 0.001

Descriptive statistics of the whole study group showed differences in maternal and neonatal factors between the two groups with a TNC count of < 150 × 10^7^ and ≥ 150 × 10^7^, respectively. The median weight of the collection bag for a successful CBU donation (≥ 150 × 10^7^ TNC) was 153 g (118–234). There were significant differences between the two groups: Higher maternal weight on admission (*p* = 0.101), higher gestational age (*p* = 0.007), first delivery (*p* = 0.007) higher estimated fetal weight (*p* < 0.001), and a higher circumference of the fetal abdomen (*p* < 0.001) were associated with a TNC count of ≥ 150 × 10^7^ cells (Table 1).

**Fig. 1:**
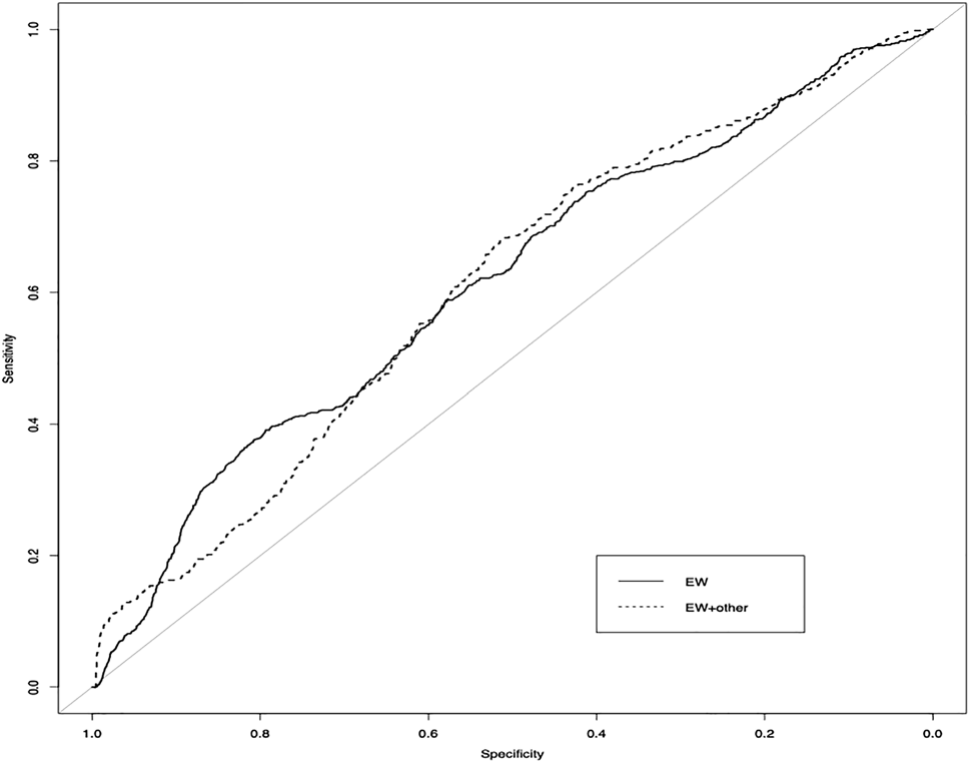
Prediction of TNC ≥ 150 × 10^7^ by estimated fetal weight and by all other prenatal factors (*n* = 594). ROC analysis showing AUC for estimated fetal weight (EW), AUC 0.62 and for all prenatal factors (EW + other) AUC 0.62, *p* = 0.98

**Fig. 2:**
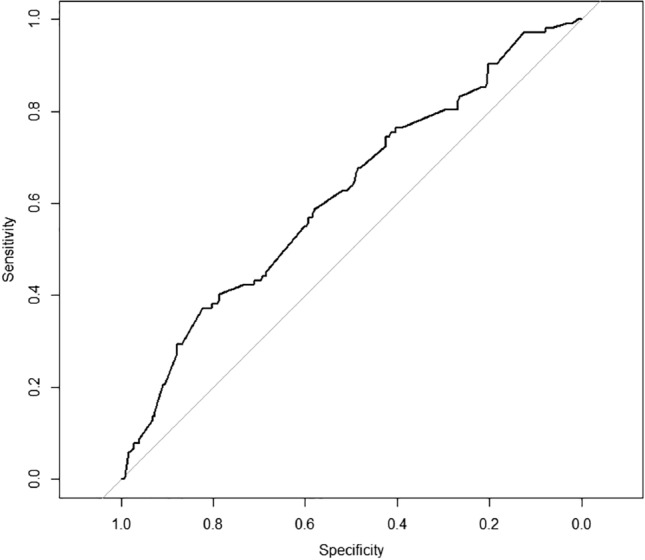
ROC analysis (AUC 0.61) for prediction of TNC ≥ 150 × 10^7^ or estimated fetal weight in women planning for a normal delivery with spontaneous onset of labor

**Fig. 3:**
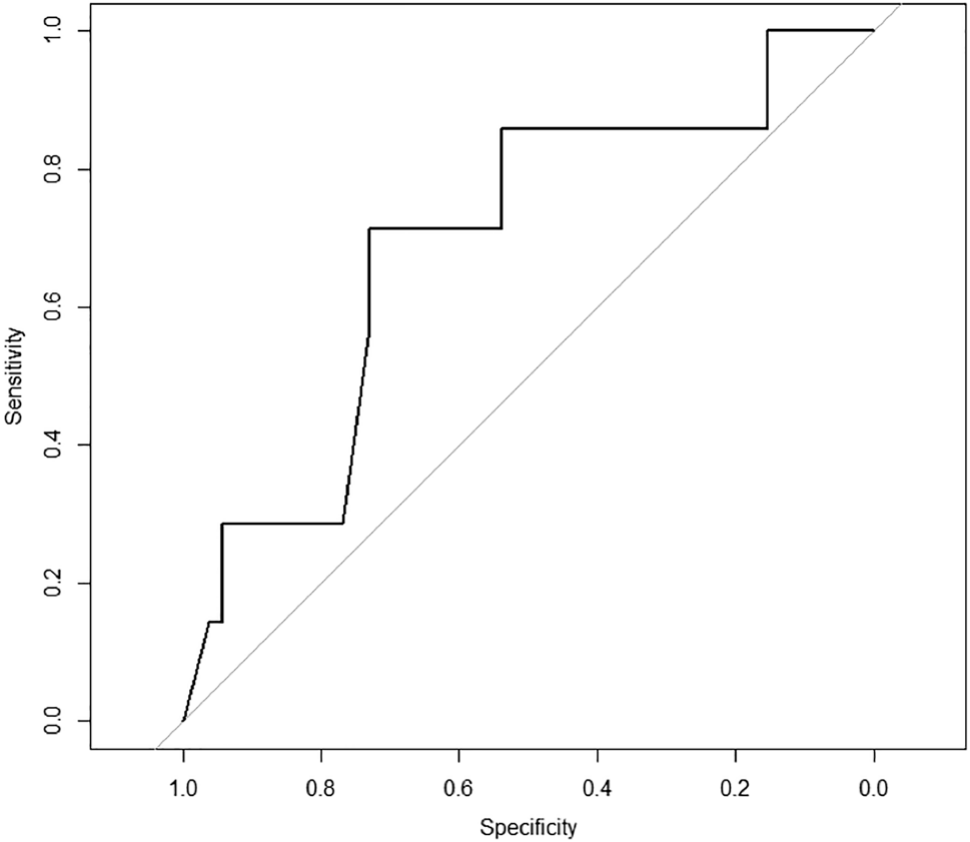
ROC analysis (AUC 0.69) for prediction of TNC ≥ 150 × 10^7^ for estimated fetal weight in women planning an elective CS ROC Analysis regarding estimated birth weight in the planned CS group (*n* = 59) AUC 0.69

In the multivariate analysis six predictors for a TNC count of ≥ 150 × 10^7^ were relevant for the whole study group (*n* = 594): parity, gestational week at delivery, maternal weight on admission, maternal height, fetal sex and estimated fetal weight. Estimated fetal weight (*p* < 0.001) and circumference of the fetal abdomen (*p* < 0.001) were the strongest predictors. Multivariable logistic regression with ten times tenfold internal cross validation was performed with all predictors and with estimated fetal birth weight alone. There was no significant difference between the AUC of all prenatal factors (AUC 0.62) and estimated fetal birth weight alone (AUC 0.62), *p* = 0.98) (Fig. 1). For women planning a trial of labor the AUC for the prediction of TNC ≥ 150 × 10^7^ was 0.61 (Fig. 2), For women planning to have a CS the AUC for the prediction of TNC ≥ 150 × 10^7^ was 0.69 (Fig. 3).

Table two presents the effect on recruitment and banking for several estimated fetal weight cut-offs (*n* = 487 complete data sets). Without a cut-off for recruitment all possible donors would be recruited but only 19.5% of units would be useful for banking. Taking estimated fetal birth weight as a selection criterion, a cut-off for recruitment at an estimated birth weight of 3200 g for women planning to undergo a trial of labor would allow 80.0% of all donors with sufficient TNC count to be recruited and 21.2% of all collected CBUs would have sufficient TNC count for banking. A cut-off of 3300 g would allow 72.6% of all donors with sufficient TNC count to be recruited and 22.8% of all collected CBUs would have sufficient TNC count for banking (Table 2). For women planning for elective CS (*n* = 59 complete data sets) an estimated birth weight of 3200 g as cut-off would allow 85.7% of all donors with sufficient TNC count to be recruited but only 16.7% of all collected CBUs would have sufficient TNC count for banking. A cut-off of 3400 g would allow 71.4% of all donors with sufficient TNC count to be recruited and 22.7% of all collected CBUs would have sufficient TNC count for banking (Table 3).

**Table 2:** CBU banking rates according to estimated fetal weight cut-offs in women planning a trial of labor

Estimated fetal weight	CBU (*n*)			Banking rate (%)
	Recruited	Not banked	Banked	
No cut off	487	392	95	19.5
3200 g	358	282	76	21.2
3300 g	302	233	69	22.8
3400 g	255	196	59	23.1
3500 g	214	162	52	24.3
3600 g	149	108	41	27.5
3700 g	108	72	36	33.3
3800 g	75	49	26	34.7

**Table 3:** CBU banking rates according to estimated fetal weight cut-offs in women with a planned CS

Estimated fetal weight	CBU (*n*)			Banking rate (%)
	Recruited	Not banked	Banked	
No cut off	59	52	7	11.9
3200 g	36	30	6	16.7
3300 g	27	22	5	18.5
3400 g	22	17	5	22.7
3500 g	18	14	4	22.2
3600 g	9	7	2	22.2
3700 g	8	6	2	25.0
3800 g	7	5	2	28.6

## Discussion

This is the first prospective study on predictors for the selection of donors for public CBU banks. Our study shows that estimated fetal weight can be used as single predictor for stem cell content and is as reliable as a combination of other fetal and maternal factors which are known before delivery. This simple triage mechanism can help to limit the efforts of recruitment and collection by ensuring that relevant numbers of high quality CBUs are collected and banked for future use for hematopoietic stem cell transplantation.

Since estimated fetal weight alone and the combination of all prenatal factors showed no significant difference, we propose using the single predictor of estimated fetal weight, usually performed during routine care within 2 weeks of delivery or at the entry to the delivery unit. Our data from the univariate analysis shows that the circumference of the fetal abdomen is a significant predictor in the prenatal setting. These measures are strictly correlated with potential stem cell production, which is dependent on hematopoietic activity [16]. Our study demonstrates that it can be used for prediction of stem cell content even tough random errors of fetal weight estimation are mostly reported to be larger than 7% [17]. Using a fetal weight estimation cut-off can reduce the effort of informed consent interviews and pre-collection preparations as well as post-collection work up and it has the potential to increase the efficacy of banking from 1:5 without using a cut-off to a maximum of 1:3 according to the desired banking numbers.

We confirmed the results of previous retrospective studies on maternal and prenatal factors influencing the stem cell content of CBUs. Dependent variables are gestational age, birthweight, sex of the baby, mode of delivery, umbilical arterial and venous pH and pathologic fetal heart rate tracing as well as fetal distress during labor [7, 9–12, 18]. However, factors developing after the onset of active labor cannot be used for prediction at the time of recruitment. Although the timing for consent to an CBU donation is handled differently among institutions, there seems to be a consensus that the best time to inform the expectant mothers is the prenatal period [19] and written consent should be obtained before the onset of active labor [20].

In the past years, the optimal cord clamping time has been discussed as recent studies showed that delayed cord clamping increases placental perfusion and increases neonatal blood volume and iron stores [21]. Delayed cord clamping greatly diminishes the volume and TNC count of CBUs significantly collected for a public CBU bank [22, 23]. In our study clamping of the umbilical cord within 30–60 s after delivery was performed in term neonates only according to current guidelines. Since collection of umbilical cord blood should not compromise obstetric or neonatal care or alter routine practice for the timing of umbilical cord clamping CBU donation certainly can only be recommended for healthy term babies [24].

This study was conducted in a public cord blood bank aiming at the registration and storage of umbilical cord blood with a good chance of being used for established therapies that are clinically used today (i.e. hematopoietic stem cell transplantation) [1]. In contrast private cord blood banks aim to create an insurance for the newborn for potential autologous use in future therapies that potentially might allow lower TNC counts to be sufficient [25]. Since stem cell content is likely to remain crucial, research is ongoing and these therapies remain to be fully established, private cord blood banks should adhere to the same standards as public cord blood banks. Compared to other sources of stem cells that are used for hematopoietic stem cell transplantations like bone marrow, cord blood can be easily collected but has the disadvantage of limited number of stem cells. Several attempts to increase the number of stem cells and have recently described by Broxmeyer [26] but no groundbreaking technique in the laboratories has been established so far. Therefore, the TNC count in the collected cord blood sample is crucial for the chances of being clinically used for hematopoietic stem cell transplantation and for a successful treatment of the recipient.

The TNC count of umbilical cord blood is an important factor in determining successful allogeneic hematopoietic stem cell transplantation after a minimum HLA donor-recipient match. A minimal TNC count of more than 2.5 × 10^7^/kg body weight or 150 × 10^7^ TNCs in an adult weighing 60 kg is recommended in the presence of no more than two HLA mismatches [6]. Corresponding to this recommended TNC count and to the current guidelines of our CBU bank we focused on predictors of TNC count of at least 150 × 10^7^. Since recruitment is often performed by the residents and midwives during the routine workup during prenatal counseling or at entry to the delivery unit time for recruitment and collection is crucial. We calculated the time needed for recruitment, informed consent, and the administrative procedures after a CBU donation to be approximately one hour, which could be saved if the probability of a successful CBU donation was low.

Mode of delivery and fetal distress during labor has an impact on the TNC count [9, 10, 12, 18]. According to the latest data from 150 countries, currently 18.6% of all births occur by CS [27]. The differentiation between women planning a trial of labor and women having a planned CS in recruiting for CBU donation is clinically useful since the chances of a successful donation in elective CS is 12.9% in contrast to 19.9% for planning a trial of labor according to our data. There is only a limited number of studies that have differentiated between elective CS and secondary CS. Two of our previous studies have consistently showed that elective CS had higher volume but lower TNC count as compared to secondary CS for fetal distress which had a significant higher TNC count [7, 9]. This finding is supported by this prospective study (data not shown) and is reflected in the banking rates (Table 1). It therefore makes sense to use different cut-off values between the two delivery groups regarding estimated fetal weight. We propose to use 3300 g for women planning a trial of labor and 3400 g for women having an elective CS in our unit. This proposed approach leads to an increased efficiency of recruitment from 12 to 23% for the elective CS group (Table 3) and to a moderate increase in the women electing a trial of labor from 20 to 23% (Table 2) while still allowing relevant numbers of cord blood units to be banked.

Obviously, the prediction of a successful donation before delivery with an AUC of 0.62 and 0.69 is limited as demonstrated in our study due to the fact that the course of delivery is known to have a significant influence on CBU content but is not an option for donor recruitment. However, the use of one single parameter for screening donors will improve efficacy and the cut-off for recruitment can be set according the goals of the collection centers individually.

Our data is not only useful for public CBU banks. The results are important to realize for all caregivers counseling or performing umbilical cord blood collection in the context of directed family banking, private or research use. The majority of CBUs will have TNC count that is too low for todays established methods of clinical use (i.e. hematopoietic stem cell transplantation).

In the context of donor recruitment strategies, an ideal selection criterion that could clearly differentiate between sufficient and insufficient stem cell content based on factors from the mother and the child during a routine pregnancy is not available. A combination of factors is not superior to fetal weight estimation in our study and has not been presented in other studies. Future studies could focus on strategies for “fast track” recruitment of all possible donors limiting the workload prior to CBU banking. Also, strategies of ex-vivo expansion of hematopoietic stem cells overcoming the main limitation of umbilical cord for transplantation are being investigated [28–30]. For now, fetal weight estimation by ultrasound seems to be a simple strategy that can easily be integrated into antenatal visits.

The strength of this study is the prospective study design from a center that has an established public cord blood bank for more than two decades with large experience in collection, standardized staff training and a focus on obstetrical implications of CBU collection. The limitation is the single center setting and future studies are needed to confirm the generalizability of the data. However, the results of this study are in line with previous retrospective studies and with published data from other centers.

## Conclusion

Intrapartum factors continue to play an important role for the quality of cord blood units but are not suitable for donor recruitment. This study demonstrates, that antenatal factors such as the use of sonographic fetal weight estimation within 2 weeks before delivery can effectively be used for counseling of donors and optimization of donor selection to public CBU banks. Since time and workload for recruitment and collection are crucial, this study offers a proposed cut-off for recruitment of 3300 g for women planning a trial of labor and 3400 g for women with elective CS to increase the efficacy of recruitment and collection while still allowing relevant numbers of CBUs to be banked.

## Data Availability

The collected data are stored in anonymized form and protected by password at University Hospital Basel.
